# Cell Types Used for Cultured Meat Production and the Importance of Myokines

**DOI:** 10.3390/foods10102318

**Published:** 2021-09-29

**Authors:** Sibhghatulla Shaikh, Eunju Lee, Khurshid Ahmad, Syed-Sayeed Ahmad, Heejin Chun, Jeongho Lim, Yongho Lee, Inho Choi

**Affiliations:** 1 Department of Medical Biotechnology, Yeungnam University, Gyeongsan 38541, Korea; sibhghat.88@gmail.com (S.S.); gorapadoc0315@hanmail.net (E.L.); ahmadkhursheed2008@gmail.com (K.A.); sayeedahmad4@gmail.com (S.-S.A.); po98053@gmail.com (H.C.); lim2249@naver.com (J.L.); 2 Research Institute of Cell Culture, Yeungnam University, Gyeongsan 38541, Korea; 3 Department of Biomedical Science, Daegu Catholic University, Gyeongsan 38430, Korea; ylee325@cu.ac.kr

**Keywords:** cultured meat, muscle satellite cell, myogenesis, culture medium, myokines

## Abstract

The world’s population continues to increase, meaning we require more consistent protein supply to meet demand. Despite the availability of plant-based protein alternatives, animal meat remains a popular, high-quality protein source. Research studies have focused on cultured meat (meat grown in vitro) as a safe and more efficient alternative to traditional meat. Cultured meat is produced by in vitro myogenesis, which involves the processing of muscle satellite and mature muscle cells. Meat culture efficiency is largely determined by the culture conditions, such as the cell type and cell culture medium used and the biomolecular composition. Protein production can be enhanced by providing the optimum biochemical and physical conditions for skeletal muscle cell growth, while myoblasts play important roles in skeletal muscle formation and growth. This review describes the cell types used to produce cultured meat and the biological effects of various myokines and cytokines, such as interleukin-6, leukemia inhibitory factor, interleukin-4, interleukin-15, and interleukin-1β, on skeletal muscle and myogenesis and their potential roles in cultured meat production.

## 1. Introduction

The architecture of skeletal muscle (SM) contains specific, well-defined arrangements of multinucleated contractile muscle cells (also known as muscle fibers) and associated connective tissue [1]. Artificial meat produced from the differentiation of muscle satellite cells (MSCs) in vitro is referred to as cultured meat. In addition to reducing the need for livestock, the adoption of cultured meat has huge environmental benefits [2,3]. In recent years, techniques such as stem cell isolation, ex vivo cell culture, and tissue engineering have been devised that enable the generation of MSCs and mesenchymal tissues. For example, bio-artificial muscles derived from MSCs are being investigated as possible surgical implants [4]. Furthermore, muscles produced by MSC culture are considered an excellent source of animal protein. Structural muscle cells could be used to produce meat in vitro. During the last few decades, myoblasts and MSCs have been used to produce cultured meat [5], and some MSC subsets have been shown to have greater regenerative ability and are preferred for SM tissue engineering [6].

Muscle development starts in vivo during early embryogenesis with the limited proliferation of mononucleated myoblasts, which then fuse and divide into non-proliferative multinuclear myofibers or muscle fibers [7,8]. MSCs are the postnatal counterparts of embryonic myoblasts [9]. MSCs are found between the sarcolemma and basal lamina and participate in muscle development, recovery, and regeneration [8]. MSCs proliferate and divide to form new myofibers after being activated by myogenic factors [10]. Several cell types have been studied for their ability to initiate the production of cultured meat, and MSCs have emerged as the most promising of these. MSCs play an important role in muscle regeneration after injury because they effectively differentiate into myotubes, which then transform into muscle fibers [8].

Once MSCs have been multiplied in vitro to the optimal amount for cultured meat processing, they must be differentiated into multinucleated, postmitotic muscle fibers by cell fusion. Switching MSCs to a differentiation medium initiates the first differentiation stage to myotubes in vitro, which takes about 3 to 5 days [11]. The immature myotubes produced are small, tubular structures that tend to synthesize neonatally, which are eventually arranged into nascent sarcomeres by adult isoforms of the contractile proteins actin and myosin [12].

MSC activity can be regulated using extracellular signaling molecules, which are usually present in culture medium. MSC activation and proliferation are mediated by growth factors (GFs) such as insulin-like GFs (IGF-1 and -2), fibroblast GF, hepatocyte GF, and cytokines (TNF-α, and LIF (leukemia inhibitory factor)) [13,14,15]. In the tissue engineering field, biopolymers, GFs, and enzymes are used extensively in cell cultures [16]. Myokines are proteins secreted by SM, which have been found to influence muscle physiology and to have systemic effects on other tissues and organs, including the adipose tissue, liver, and brain [17]. Immune-derived cytokines influence SM development, organ formation, regeneration, and wasting, although muscle fibers also generate and secrete cytokines and myokines. TNF-α and interleukin-6 (IL-6) are the most effective immune-derived cytokines in terms of activating MSCs after muscle damage, whereas IL-8 coordinates growth and regeneration and IL-15 controls muscle hypertrophy [18]. In this review, we describe the effects of various myokines and cytokines on SM function and myogenesis and their possible roles in cultured meat production.

## 2. Status of Cultured Meat

Since the first cultured beef hamburger was made in 2013, dozens of companies have entered the cultured meat sector, and a variety of product species, including chicken, beef, pork, and seafood, are now being developed. By the end of 2020, about 60 early-stage companies concentrating on cultured meat end-products and raw materials throughout the value chain had been established worldwide, with more than half of them having launched within the last two years [19,20]. These companies are geographically distributed across 19 countries and five continents, with 37% in North America, 25% in Asia, and 21% in Europe. The total amount of money invested in cultured meat companies from 2016 to 2020 was estimated to be over $460 million, with more than three-quarters of that amount (more than $350 million) being invested in 2019 and 2020. Memphis Meat has raised nearly $200 million to take the lead, while Mosa Meat has raised over $85 million. In terms of product interest, 28% of cultured meat companies are interested in cultured beef and pork, while 12% are interested in seafood, 10% in poultry, and 28% in raw materials or equipment used in the production process [21].

## 3. Cell Types for Cultured Meat

One or more starting cell populations are used to begin the cultured meat production process, and thus, initial cell populations may be homogenous or heterogeneous. Despite the complexities of meat, it is currently thought that MSCs and adipocytes are essential components. The suitability of starter cells for culture is dependent on their ability to self-renew and differentiate in a setting in which other animal-derived elements, such as serum, are reduced. The discovery of stem cells paved the way for in vitro cell production and the development of cultured meat. A biopsy from a live animal can be used to sample stem cells, which can then be expanded in vitro to produce substantial cell numbers [22]. Based on the types of stem cells isolated, these cells can be activated to differentiate into muscle or fat cells. MSCs are a reliable cell source for SM recovery in vivo [23], and their self-renewal ability maintains stem cell populations and the productions of large numbers of myogenic cells, which proliferate, divide, fuse, and contribute to the development of new myofibers [8]. The first cultured meat hamburger prototype was produced by amplifying the myoblast progeny of MSCs [22]. Multipotent progenitor cells originating from porcine SM have a higher doubling capacity than MSCs making them a better cell source for cultured meat; however, these cells require costly recombinant GFs and do not differentiate into SM fibers as efficiently as MSCs [24]. Pluripotent stem cells (derived from non-muscle sources; PSCs) can be isolated from a variety of domesticated animals and used as myogenic cell sources for cultured meat. Chemically and genetically engineered porcine PSCs have recently been transformed into myogenic cells capable of differentiating into embryonic muscle fibers [25]. While PSCs are an appealing possible cell source, any cultured meat produced from them must be identified as genetically modified and undergo rigorous safety testing.

Muscle progenitor cells come in a wide variety of forms. Within muscle, the different tissue types (nerves, blood vessels, and adipose and connective tissues) necessitate the presence of a large number of progenitor cells with various lineage differentiation plasticity levels [26]; thus, it is important that the populations of progenitor cells be differentiated. Biomolecular marker expression profiles and cell differentiation capacity levels have been used to classify muscle progenitor cell types. The majority of MSCs have been identified in humans and mice, although as porcine organs are functionally and anatomically almost identical to those of humans, porcine MSCs are viewed as useful raw materials in regenerative therapy research [24].

## 4. Roles of Myokines in Skeletal Muscle

### 4.1. Interleukin-6 (IL-6)

IL-6 is a multifunctional cytokine involved in immune response modulation and coordination [27], with growing evidence suggesting that muscle cells are a suitable source of IL-6 [28]. Human primary myoblasts and murine C2C12 myoblast cells also produce IL-6 in vitro [29,30]. In cultured C2C12 cells, IL-6 mRNA knockdown reduces the expression of muscle-specific genes [31], which demonstrates that this cytokine has a myogenic function. Furthermore, local IL-6 production increases MSC activation and promotes myotube regeneration [32], while treatment with IL-6 has been shown to increase MSC proliferation by regulating the cyclin D1 and c-myc genes. In addition, IL-6 deficiency inhibited MSC proliferation and myonuclear accretion in preexisting myofibers by impairing STAT3 activation [33]. In differentiating C2C12 cells, STAT3 or IL-6 mRNA knockdown reduced the expression levels of myogenin and myosin heavy-chain (MyHC) IIb, causing myotube fusion to be disrupted. Moreover, myoblasts derived from IL-6 null mice exhibited reduced fusion potential in vitro, which confirmed the participation of IL-6 in myogenic differentiation [34]. In monocytes and macrophages, IL-6 expression and activation of the STAT3 signaling pathway were key mediators of macrophage migration and myoblast proliferation during muscle regeneration [35]. The expression of IL-6 receptors was reduced after 24 h of treatment with IL-6 at high concentrations but increased at low concentrations. Low IL-6 concentrations stimulate proliferation, whereas high concentrations promote differentiation, and both processes are regulated by components of the JAK/STAT/SOCS pathway [36]. Altogether, these findings suggest that IL-6 functions as an important regulator of myogenesis by acting during myoblast proliferation and differentiation.

### 4.2. Leukemia Inhibitory Factor (LIF)

LIF is a member of the IL-6 cytokine family, is secreted by a variety of cell types, and has biological effects that depend on the target tissue type and cell lineage. Local LIF formation by regenerating muscle and other cells in muscle is essential for muscle regeneration [37,38]. The expression of LIF mRNA was triggered by high-intensity resistance exercise capable of inducing SM hypertrophy. It has been demonstrated that LIF influences myoblast proliferation, differentiation, and regeneration [39,40,41]. Exogenous LIF stimulated human myoblast proliferation by inducing the transcription factors JunB and c-Myc, while siRNA knockdown of endogenous LIF receptor inhibited myoblast proliferation [42]. In addition, LIF was identified as the upstream component that stimulated myoblast differentiation by activating the JAK2/STAT3 pathway [43], while LIF was found to be important for the survival of embryonic stem cells and to promote myoblast proliferation in mice and rats [15,44].

### 4.3. Interleukin-4 (IL-4)

The transcription factor NFATc2 (nuclear factor of activated T cells) is required for cell growth and regulates myoblast fusion at a particular stage of myogenesis after initial myotube development. NFATc2 induces IL-4, which facilitates cell fusion and muscle development. Furthermore, IL-4 regulates cell fusion by acting on myoblasts rather than myotubes via IL-4 receptor (IL-4R). Myoblasts and myotubes also express IL-4Rα, which is necessary for muscle development, while myoblasts lacking IL-4Rα are not recruited by IL-4-secreting nascent myotubes and develop normally, although are smaller, and myotubes have lower myonuclear numbers. IL-4 promotes the fusion of myoblasts to nascent myotubes during growth and regeneration [45] and IL-4 treatment enhances myogenic cell migration and promotes mononuclear cell interactions with nascent myotubes [46]. Recent studies showed that IL-4 stimulates myogenesis by increasing the expression levels of myogenic transcription factors [47] and that the presence of adipose-tissue-derived stromal cells can increase MSC regeneration, which was promoted by pretreating IL-4 or SDF-1 [48].

### 4.4. Interleukin-15 (IL-15)

IL-15 is expressed at higher mRNA and protein levels in SM than in other tissues [49,50,51] and has been linked to the interaction between muscle and adipose tissues [52]. Furthermore, it has been demonstrated that adipose tissue deposition can be modulated by muscle-derived IL-15 [53]. IL-15 stimulates the accumulation of MyHC protein in differentiated myotubes in human SM cell cultures, which suggests that IL-15 acts as an anabolic agent during muscle development [54]. In addition, quantitative real-time PCR revealed that C2C12 cells express IL-15 and that IL-15 mRNA levels are upregulated more than 10-fold in differentiated rather than undifferentiated cells [55]. In cultured skeletal myotubes, IL-15 stimulated protein synthesis while inhibiting protein degradation [55,56], while IL-15 secreted by myotubes, derived from human SM, promoted human myotube development by increasing the expression levels of myogenic marker genes [57].

### 4.5. Interleukin-1β (IL-1β)

After SM injury, the surrounding environment has substantial impacts on MSC function. Immune cell infiltration dominates the extracellular space in damaged areas causing cytokine levels to rise, while in mice tissue it was reported that 5 days after injury, IL-1β expression was increased ~20-fold. Furthermore, elevated IL-1β expression stimulates MSC proliferation by activating NF-κB [58].

### 4.6. Effects of Cytokines in Combination

Immune cell infiltration increases the release of proinflammatory cytokines such as interleukins, TNFs, and interferons in injured regions after muscle injury. Based on this finding, Fu et al. [59] cultured MSCs long-term in vitro in the presence of T-cell released IL-1α, IL-13, TNF-α, and IFN-γ. In combination, these four cytokines enabled the in vitro maintenance of MSCs in an undifferentiated state over 20 passages [59]. The effects of cytokines on SM function and myogenesis are summarized in Table 1.

### 4.7. Myostatin

Myostatin (MSTN) was the first myokine discovered in 1997. MSTN belongs to the transforming growth factor-β family and negatively regulates SM mass [60]. Plasma MSTN levels were found to decrease dramatically within 24 h of exercise in healthy young men and to be positively correlated with plasma IL-6 levels [61]. On the other hand, serum MSTN levels were found to increase during aerobic activity in patients with spinal cord injury [62]. MSTN gene-inactivating mutations are linked to enhanced muscle mass and reduced fat mass, while MSTN transgenic mice that overexpress MSTN in SM have reduced muscle mass and enhanced fat mass [63,64]. The doubling of muscle mass in cattle, sheep, dogs, and humans with natural mutations in the MSTN gene suggests that the function of MSTN has been conserved across species [60,65,66,67,68]. These results raised the prospect that inhibiting MSTN activity may have significant therapeutic and agricultural implications.

Several extracellular matrix (ECM) proteins, including fibromodulin (FMOD), decorin, fibronectin (FN), and laminins, have been shown to bind to MSTN and control its function [69,70]. Previously, we explored a number of ECM proteins, such as FMOD [71,72], matrix gla protein [73], and dermatopontin [74], which play important roles in the regulation of myogenesis. MSTN inhibits the proliferation and differentiation of MSCs and thereby inhibits muscle development [75]. We found that FMOD circumvents the inhibitory effects of MSTN and regulates myogenesis by enhancing the expression levels of myogenic marker genes. Furthermore, an investigation of the protein–protein interaction between FMOD and MSTN showed that FMOD decreases MSTN sensitivity to activin type IIB receptor (ACVRIIB, MSTN receptor) binding affinity [71]. Recently, we reported that *Glycyrrhiza uralensis* (a medicinal herb) inhibits MSTN expression and promotes myogenesis [76].

### 4.8. Irisin

The myokine irisin is a cleaved portion of the transmembrane protein fibronectin type III domain-containing 5 (FNDC5) [77], and a possible mediator of the positive impact of exercise [78,79]. FNDC5 expression is stimulated by peroxisome proliferator-activated receptor gamma coactivator-1-alpha (PGC-1α) expression in muscle and leads to the production of brown fat-like growth of white fat cells and enhanced thermogenesis [80]. Although exercise-induced increase in blood irisin levels is controversial [81], several studies have reported *FNDC5* expression is enhanced in animal models and humans during exercise [82,83,84,85], which reintroduce interest in exercise-stimulated myokines. Myotubes exposed to recombinant irisin for 24 h exhibited enhanced expressions of mitochondrial-specific transcription factors like PGC-1α and mitochondrial transcription factor A, both of which are involved in increasing myocytes and adipocytes mitochondrial contents and oxygen intake [86]. Irisin was reported to activate IL-6 signaling in mice resulting in SM hypertrophy and reduced denervation-induced atrophy [87]. In another study, irisin caused hypertrophy by activating MSCs and increasing protein synthesis [87]. These findings pave the way for future studies on irisin in the context of muscle atrophy

### 4.9. Myonectin

Myonectin belongs to the CTRP (C1q/TNF-related protein) family and is a newly discovered nutrient-responsive myokine produced by SMs [88,89,90]. Myonectin protein is released into the bloodstream as a result of muscle contractions and functions similarly to insulin by facilitating cellular fatty acid uptake by increasing the expression levels of fatty acid transport genes [88,90]. Recombinant myonectin inhibits LC3-dependent autophagosome production, inhibits the production of other autophagy-related genes in the rodent liver and in cultured hepatocytes, and suppresses starvation-stimulated autophagy. Inhibition of the PI3K/Akt/mTOR signaling pathway prevented myonectin-induced suppression of autophagy [91], which is considered to be one of the mechanisms responsible for muscle atrophy [92]. Furthermore, the PI3K/Akt signaling pathway plays a role in anabolic responses. These results demonstrate that myonectin appears to play a key role in enhancing muscle mass by increasing protein synthesis and preventing protein degradation.

Muscle mitochondrial content is a key determinant of muscle form and function. In rat skeletal myocytes, mtDNA depletion results in a significant increase in myonectin, which enhances glucose intake and fatty acid oxidation by activating the AMPK signaling pathway [93,94]. Myonectin is expressed at a higher frequency in oxidative slow-twitch muscle fibers than in glycolytic fast-twitch muscle fibers, which suggests it might be involved in mitochondrial biogenesis and cellular energy-sensing [90].

### 4.10. Decorin

**Figure 1:**
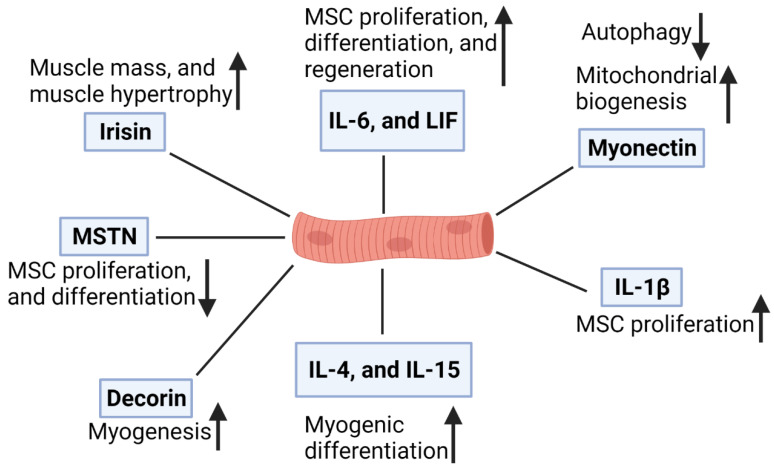
Function of myokines in skeletal muscle.

Decorin (DCN) is a myokine secreted by SMs during contraction and is involved in muscle development [95]. DCN binds to MSTN and prevents its antimyogenic effects by inactivating it in a zinc-dependent manner [96]. In murine SMs in vivo, DCN overexpression facilitates the expression of the promyogenic factor Mighty, which is widely expressed, although it seems to be inhibited in SMs by MSTN [95]. In addition, DCN overexpression enhanced the expression levels of Myod1 and follistatin but reduced the expression levels of muscle-atrophy-related genes (atrogin1 and MuRF1) [95]. Myokine functions are presented in Figure 1.

## 5. Advantages and Disadvantages of Cultured Meat

Foodborne diseases, antibiotic-resistant strains, resource use, farm animal welfare, and the environmental consequences of raising livestock, such as pollution from their excrement and massive methane emissions contributing to global warming, are just a few of the serious concerns linked with conventional meat production systems, about which consumers have expressed their dissatisfaction [97]. Given the significant negative consequences of meat production on the environment and human health, cultured meat production, a technique that has the potential to change human existence, represents a feasible alternative. Cultured meat production may provide health and environmental benefits by decreasing pollutants, water usage, and land use connected with existing meat production methods [98,99]; therefore, cultured meat production techniques show a lot of potential for the environment.

The cell culture technique has significant benefits in terms of cell homogeneity, showing nearly complete control over myogenesis. In addition, MSC can increase by at least 20 times when cultured under ideal conditions. As such, a significant amount of muscle fibers may be obtained from a very small number of isolated cells. Future efforts should be focused on somatic cell nuclear transfer into oocytes (cloning), embryonic stem cells, or the ectopic expression of specified factors in inducible PSCs as constantly proliferating cells capable of effectively differentiating into myotubes and muscle fibers [100,101,102]. Adjustments to the texture, taste, and flavor of the cultured meat are also possible.

Cultured meats may have some difficulty competing with regular meats in terms of their color and appearance; therefore, to improve the appearance and flavor of the cultured meat products, new meat processing methods must be developed. Scaffolds made from natural and edible biomaterials, such as collagen, which allow for 3-D tissue development and complex meat structures, have also been suggested and attempted [103].

The cultured meat production method may disconnect us from nature and animals and may be a step toward sustainable urbanization. Cultured meat products fit our growing dependence on technology, although there is concern that this may lead to an increasing disconnect from nature [104]. The primary potential barrier is the high cost of cultured meat, despite the fact that large-scale manufacturing and market penetration are generally linked with substantial price decreases. Cultured meat production on a large scale is only possible if a reasonably low-cost technique is developed that produces a product that is qualitatively comparable to existing meat products and is subsidized by the government in the same way that other agribusinesses are subsidized [98,105].

## 6. Future Prospects

**Figure 2:**
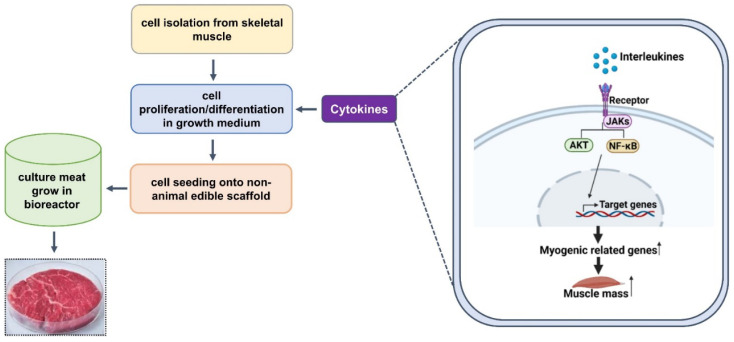
The cultured meat production process and the putative roles of myokines.

MSCs are the most important components of cultured meat, the production of which requires their multiplication to specific progenitor cells and differentiation into SM cells. The quantity of cultured meat produced is determined by the proliferation of MSCs, and higher cell growth rates are accomplished by improving the efficiency of cell doubling processes. Differentiation is a crucial step in achieving the desired characteristics of cultured meat, although proliferation and differentiation media preferences are variable and dependent on their composition. Furthermore, as the process progresses from the proliferation to the differentiation and maturation phases, the changing needs of cells may require culture media adjustments. Although most of the components necessary for proliferation and differentiation in commonly used media are identical, GFs and compounds that drive differentiation are critical for the differentiation and maturation phases. Fetal bovine serum (FBS) contains a high concentration of GFs and promotes cell growth; however, although FBS is useful for cell culture, it has several limitations. The development of culture media for the large-scale production of high-quality cultured meat products remains a huge challenge. This review shows that various myokines are involved in MSC activation, proliferation, and differentiation, and that IL-15 stimulates the accumulation of MyHC protein (a putative biomarker of final myogenic differentiation) in differentiated myotubes. We anticipate that myokines are likely to be important components of serum-free media that promote cell growth and control cell activity during cultured meat production (Figure 2).

## 7. Conclusions

Cultured meat is a good source of protein and is being studied as a safe and efficient alternative to regular meat. It also has the potential to provide an inexhaustible supply of dietary protein in a cost-effective and environmentally friendly manner. Acceptance of cultured meat is said to be dependent on its efficiency and ability to mimic traditional meat properties. Current technologies such as stem cells, tissue engineering, and tissue culture make it possible to produce cultured meat; however, more advancements in this field are required, as well as extensive investigation of the economic effectiveness of the technology and the related ethical and societal concerns, before successful large-scale manufacturing can be achieved.

## Figures and Tables

**Table 1:** Role of cytokines in myogenesis.

S. No.	Cytokines	Role in Myogenesis	References
1.	IL-6	Promotes MSC proliferation.	[33]
		Induces myogenic differentiation.	[34,36]
		Contribute to muscle regeneration.	[35]
2.	LIF	Stimulate myogenic differentiation.	[43]
		Control the proliferation of MSC.	[42]
		Contribute to muscle regeneration.	[37,38]
3.	IL-4	Act as a myoblast recruitment factor during muscle growth and control myoblast fusion with myotubes.	[45]
		Stimulate myogenic differentiation.	[47]
4.	IL-15	Expressed more in differentiated myotubes than undifferentiated myoblasts.	[55]
		Act as anabolic factor capable to increase MyHC in differentiating MSCs.	[54]
		Stimulate myogenic differentiation.	[56]
5.	IL-1β	Increase MSC proliferation.	[58]
6.	IL-1α	Combination of four cytokines (IL-1α, IL-13, TNF-α, and IFN-γ) allowed in vitro maintenance of MSC in an undifferentiated state over 20 passages.	[59]
	IL-13		
	TNF-α		
	IFN-γ		
